# Changes in Nursing Students' Clinical Handover Performance Following ISBAR‐Structured Handover Simulation Teaching: A Mixed‐Methods Study

**DOI:** 10.1002/nop2.70955

**Published:** 2026-10-26

**Authors:** Jui‐Chiung Sun, Min‐Yu Chang, Yi‐Chun Tsai

**Affiliations:** ^1^ Department of Nursing Chang Gung University of Science and Technology Taoyuan Taiwan; ^2^ Department of Nursing New Taipei Municipal TuCheng Hospital (Built and Operated by Chang Gung Medical Foundation) New Taipei Taiwan

**Keywords:** ISBAR, mixed‐methods research, nursing handover, obstetric nursing education

## Abstract

**Background:**

Clinical handover is critical for patient safety, yet nursing students frequently lack structured training, making it difficult to convey essential information accurately.

**Aim:**

To examine changes in students' communication clarity, confidence and learning experiences following ISBAR (Introduction, Situation, Background, Assessment, Recommendation) simulation‐based teaching.

**Methods:**

A mixed‐methods design with 57 nursing students combined a single‐group pretest–posttest study and focus group interviews. Data were collected from questionnaires and OSCE (Objective structured clinical examination), and analysed using SPSS 28.0 and content analysis.

**Results:**

Following the ISBAR‐structured handover simulation intervention, post‐intervention scores for communication clarity and confidence were significantly higher than pre‐intervention scores. Qualitative analysis identified four themes: (1) mastering the handover framework to enhance organized expression; (2) scenario simulation strengthening clinical reasoning and adaptability; (3) peer feedback and reflection promoting self‐awareness; and (4) progressive practice building handover confidence.

**Conclusions:**

Communication clarity and confidence improved after ISBAR simulation‐based teaching. Because the design was single‐group and uncontrolled, these changes cannot be attributed exclusively to the intervention; controlled studies are needed.

**Implications for Nursing:**

Incorporating this model into specialist nursing curricula—particularly in high‐risk areas such as obstetrics—through scenario‐based practice is recommended to strengthen communication quality; effects on patient‐safety outcomes were not assessed and warrant future evaluation.

**Reporting Method:**

This study adhered to EQUATOR guidelines and used COREQ for qualitative reporting.

**Patient Contribution:**

No patient or public contribution.

## 1 Introduction

Clinical handover is a fundamental means by which nursing professionals communicate with other members of the healthcare team, and represents a core skill for nursing students. It constitutes one of the most critical steps in a patient's care journey; its core function is to enhance the effectiveness of the incoming clinician's subsequent actions, ensuring continuity of care and patient safety (Burgess et al. 2020). As central professionals in patient care, nurses maintain the most continuous contact with patients, and any lapse in handover may give rise to patient safety concerns (Fahajan et al. 2023). Omissions of critical details and inaccuracies during handover have been identified globally as a leading cause of medical errors and adverse outcomes (Le et al. 2023; Maluenda Martinez et al. 2025). Recognizing the imperative for safety, the World Health Organization and other international health institutions have jointly investigated actionable structured handover tools to prevent the omission of critical information during handover and communication processes, thereby reducing medical errors (Shahid and Thomas 2018).

Despite the importance of handover to safe nursing practice, structured handover training rarely appears in nursing education curricula (Collins 2014; Le et al. 2023). Research has found that nursing students and newly registered nurses frequently find handover challenging, owing to limited practice in communication and self‐efficacy; they experience particular frustration with clinical handover (Chung et al. 2022). This is compounded by the fact that students on clinical placements are largely limited to observation rather than practice, making it difficult to develop communicative competence through hands‐on clinical experience (Johnston et al. 2017). This structural deficit is especially consequential in specialist fields such as obstetric nursing, where the pace of patient care, complexity and emotional intensity are high, making accurate and structured handover critically important.

ISBAR (Introduction, Situation, Background, Assessment, Recommendation) is the most internationally recognized tool; its five components guide nurses in transmitting and receiving cognition about a patient's current condition (Finnigan et al. 2010). Simulation‐based teaching is grounded in Kolb's (1984) experiential learning theory and is consistent with Miller's (1990) pyramid of clinical competence. It provides a controlled and repeatable environment in which nursing students can practice the ISBAR‐structured handover process before entering clinical practice, enhancing communication clarity and confidence through repeated reflective practice. The Objective Structured Clinical Examination (OSCE) is an evidence‐based and reliable tool that provides a standardized, objective method for assessing students' handover competence—capability that self‐report scales alone cannot provide (Bagnasco et al. 2016; Burgess et al. 2020).

Although considerable evidence documents outcomes of ISBAR‐structured handover simulation teaching in general nursing contexts, three notable gaps remain. First, only a small number of studies have examined ISBAR simulation teaching in obstetrics—a field in which unambiguous communicative competence is essential. Second, most studies report either quantitative or qualitative outcomes, but not mixed‐methods designs capable of simultaneously capturing measurable competence outcomes and students' authentic learning experiences. Finally, the use of OSCE as an objective assessment instrument specifically for handover communication remains underexplored (Maluenda Martinez et al. 2025). Addressing these gaps, this study uses an obstetric simulation and practicum course as its context to examine changes in nursing students' clinical handover performance following ISBAR‐structured handover simulation teaching. The purpose is to equip students enrolled in the course to engage in authentic handover processes—participating in simulation teaching, reflecting internally and applying knowledge—thereby cultivating communicative skills with healthcare professionals in preparation for future clinical practice.

### 1.1 Literature Review

Clinical handover refers to structured communication of patient information and professional judgement between nursing staff across shifts, units, or professions; its quality directly affects patient safety. Incomplete or inaccurate handover has been identified as a significant contributing factor to medical errors and patient harm (Burgess et al. 2020; Fahajan et al. 2023; Le et al. 2023; Maluenda Martinez et al. 2025; Vanderzwan et al. 2023). To enhance patient safety, international organizations recommend the use of standardized handover tools; ISBAR has been recognized for its high rigour and credibility and is widely applied in clinical and nursing education settings (Finnigan et al. 2010; Marshall et al. 2009; Shahid and Thomas 2018). The ISBAR framework (Figure 1) guides both the sender and receiver of handover to communicate systematically according to the patient's condition, helping to reduce the omission of critical information, eliminate irrelevant content and enhance continuity of care. Empirical research further demonstrates that ISBAR simulation‐based teaching significantly improves nursing students' handover communication quality, supporting its value in both clinical and educational applications (Finnigan et al. 2010; Noh and Park 2022).

**FIGURE 1:**
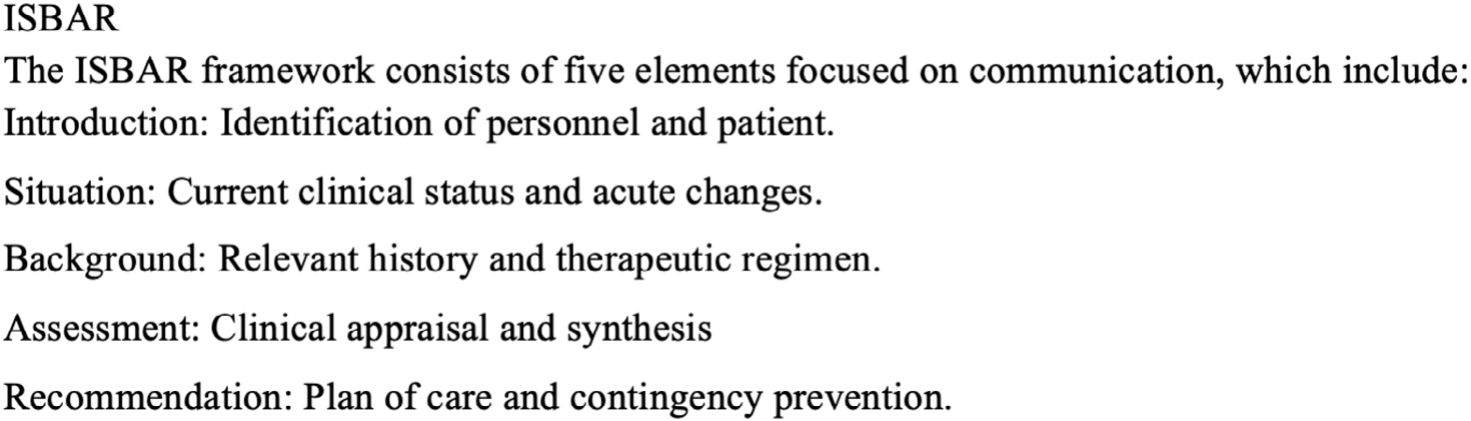
ISBAR framework.

Communication is a core competency in nursing education; students must develop effective information‐transfer skills through both classroom and clinical training. However, insufficient opportunities for hands‐on practice have led curricula to progressively incorporate handover teaching (American Association of Colleges of Nursing 2021; Kim et al. 2016; Yun et al. 2023). Research indicates that nursing students, lacking handover practice, frequently feel unfamiliar with handover, lack confidence and feel anxious upon entering clinical settings; constrained to an observational role in practice, they find it difficult to build self‐efficacy. In contrast, ISBAR‐structured simulation handover, role‐play and scenario simulation have been shown to effectively improve communication ability, confidence and learning satisfaction (Collins 2014; Johnston et al. 2017; Kostiuk 2015; Le et al. 2023; Yu and Kang 2015).

This pedagogical strategy is consistent with Kolb's (1984) experiential learning theory and Miller's (1990) framework of clinical competence, which serve as the theoretical basis for this study's intervention (Kolb 1984; Miller 1990; Uhm et al. 2019). Related research has demonstrated that SBAR/ISBAR‐structured simulation teaching enhances nursing students' communicative ability, clinical reasoning and learning satisfaction (Chung et al. 2022; Kim et al. 2016; Kostiuk 2015; Marshall et al. 2009). Furthermore, video‐assisted debriefing and structured discussion promote students' reflective capacity and understanding of clinical communication, supporting the application of ISBAR skills in complex contexts; peer feedback encourages critical thinking and self‐awareness, strengthening bidirectional communication—an aspect often neglected in traditional handover teaching (Burgess et al. 2020; Johnston‐Hanson 2012).

Clinical assessment constitutes an important component of nursing education, OSCE is regarded as standardized, objective and feedback‐oriented, capable of effectively evaluating students' clinical skills performance (Bagnasco et al. 2016; Clarke et al. 2011; Harden et al. 1975; Selim et al. 2012). Through standardized patient scenarios, OSCE can simulate authentic clinical interactions, promoting students' integration of theory and practice, enhancing clinical confidence and adaptability and reducing pre‐clinical anxiety and frustration (Barry et al. 2012; Chen et al. 2021; Mitchell et al. 2009). OSCE also helps identify gaps in students' care knowledge and reinforce positive professional attitudes (Sola et al. 2017). Although this is a widely accepted tool, it is not commonly applied to the assessment of nurses' handover communication. This reveals a clear gap: handover is inherently a performance skill requiring real‐time clinical reasoning, structured verbal communication and appropriate team response—precisely the competencies that the OSCE format can capture. Burgess et al. (2020) emphasize that using OSCE to assess ISBAR handover quality allows educators to obtain objective, criterion‐referenced data in a controlled environment. It is the responsibility of nursing education to guide students in learning ISBAR‐structured handover skills; if students are expected to deliver appropriate handovers in clinical work, they must experience that learning throughout their education (Yun et al. 2023).

## 2 Methods

### 2.1 Aim of the Study

To examine changes in nursing students' clinical handover communication clarity, confidence, learning experiences and perceptions following ISBAR‐structured handover simulation teaching in the obstetric simulation and practicum course.

### 2.2 Research Design

A mixed‐methods design was employed, comprising a single‐group pre‐post study and focus group interviews. The quantitative component utilized a pre‐experimental one‐group pretest‐posttest design; the qualitative component encompassed focus group interviews, learning reflection feedback forms and the researcher's teaching reflection records.

### 2.3 Participants and Setting

Convenience sampling was used to recruit participants from the nursing department of a university of technology in northern Taiwan. As the obstetric simulation and practicum course was an elective course, all 57 students enrolled in the course during the study period were eligible and invited to participate; the sample therefore represented the entire population of students enrolled in the course rather than a subset of the accessible population. Eligibility criteria were: (1) a third‐year student in the 4‐year nursing program; and (2) being enrolled in the elective obstetric simulation and practicum course. Written informed consent, obtained after a clear explanation of the study purpose and procedures, was a separate ethical requirement applied to all eligible students prior to enrolment, rather than an eligibility criterion in itself. Before the course orientation, the principal investigator explained the study's purpose, content, procedures and participant protections to all students. A priori sample size estimation was conducted using G*Power 3.1.9.7 for a paired‐samples *t*‐test (effect size ***d***_**z**_ = 0.43, *α* = 0.05, power = 0.80), yielding a minimum required sample size of 45. Accounting for an anticipated attrition rate of 20%, a minimum of 54 participants was targeted. A total of 57 nursing students were enrolled as study participants; no participants withdrew, and the final sample exceeded the minimum required sample size. Total instructional hours were 36. Teaching settings included the obstetric demonstration classroom, return demonstration practice room, scenario simulation room and OSCE examination venue. Prior to commencing the ISBAR‐structured handover simulation program, researchers contacted students by email to explain the study purpose and group procedures, and to invite voluntary, confidential participation in focus group interviews to be held after all course assessments had been completed (Week 18). A total of 26 students volunteered to participate in focus group interviews; the remaining 31 did not volunteer.

### 2.4 Research Intervention

The total intervention period spanned 5 weeks (Weeks 10–14), comprising 10 contact hours (Figure 2). Of the 36 total instructional hours for the course (Section 2.3), 10 h constituted the ISBAR‐structured intervention reported here; the remaining 26 h were devoted to other obstetric simulation and practicum course content not specific to the ISBAR module. The intervention included the following phases: (Know/Know How): ISBAR‐structured handover didactic instruction and expert modelling (2 h), covering definitions, significance and clinical application (Shows How): Role‐play—antenatal assessment and handover practice (2 h), in which students were grouped to assume roles of the obstetric patient, student nurse and senior nurse; each student rotated through different roles to ensure that all experienced both sender and receiver perspectives. Scenario Simulation—postpartum assessment and handover for normal spontaneous delivery and caesarean section (4 h), incorporating standardized patients (SP) and video‐assisted debriefing. All SPs received standardized training to ensure consistency across groups. Following each scenario, the principal instructor conducted approximately 20–30 min of structured video‐assisted debriefing, actively encouraging peer feedback, guiding students to review their handover performance, identify areas for improvement and connect the simulation experience to authentic clinical contexts. OSCE Assessment (2 h): two clinical examination stations—‘Antenatal Assessment’ and ‘Postnatal Assessment’—were established; each student was required to complete both stations individually. This served as the summative learning activity of the course and the primary objective performance assessment instrument of the study. The 57 students were divided into eight practice groups of six to eight members each, with all eight groups practicing simultaneously at parallel simulation stations. Each scenario comprised a 15–20‐min pre‐briefing, an 8–10‐min scenario enactment, and a 20–30‐min debriefing. Within each group, students rotated through both sender and receiver roles, so that every student actively performed the ISBAR handover in each scenario; no student served solely as an observer. Each of the eight parallel groups was facilitated by one designated faculty facilitator (ratio 1:1). All facilitators were registered nurses holding a master's degree or above, with a mean of 5 years of obstetric clinical experience, and all completed an 8‐h standardization workshop covering the scenario scripts, the debriefing protocol and the OSCE rubric. Consistency across groups was maintained through a written facilitator manual, identical scenario materials, standardized patients trained to a common script, and a facilitator review meeting after each session. Practice‐phase feedback was formative and unscored; summative assessment was confined to the OSCE. The OSCE was conducted in person by four trained assessors in four station rooms operating in parallel (two Antenatal Assessment rooms and two Postnatal Assessment rooms). Each room had a trained standardized patient and one assessor, who scored performances in real time using the same rubric (Section 2.5). Each station slot lasted 10 min, including changeover, so each student was examined in two slots (20 min in total). The 57 students belonged to two intact classes (Class A, *n* = 32; Class B, *n* = 25), and each class was examined in a separate session, with students rotating through the four rooms in successive 10‐min slots. The 2 contact hours listed for the OSCE phase in Figure 2 denote scheduled student contact time allocated to this phase in the course schedule, not the total assessment operating time. Each student was examined for 20 min (two 10‐min slots; each performance typically lasted 7–8 min). Because double‐scoring required the two designated assessors to observe 48 station performances (24 students × 2 stations) together in sequential 10‐min slots (480 min in total), while the other two assessors continued single‐scoring in their own rooms, the total assessment operating time exceeded the scheduled contact hours: Class A was examined on the first OSCE day of Week 14 from 12:30 to 17:30 (approximately 5 h), and Class B on the second OSCE day from 12:30 to 16:30 (approximately 4 h). Supplementary S1 provides the OSCE schedule.

**FIGURE 2:**
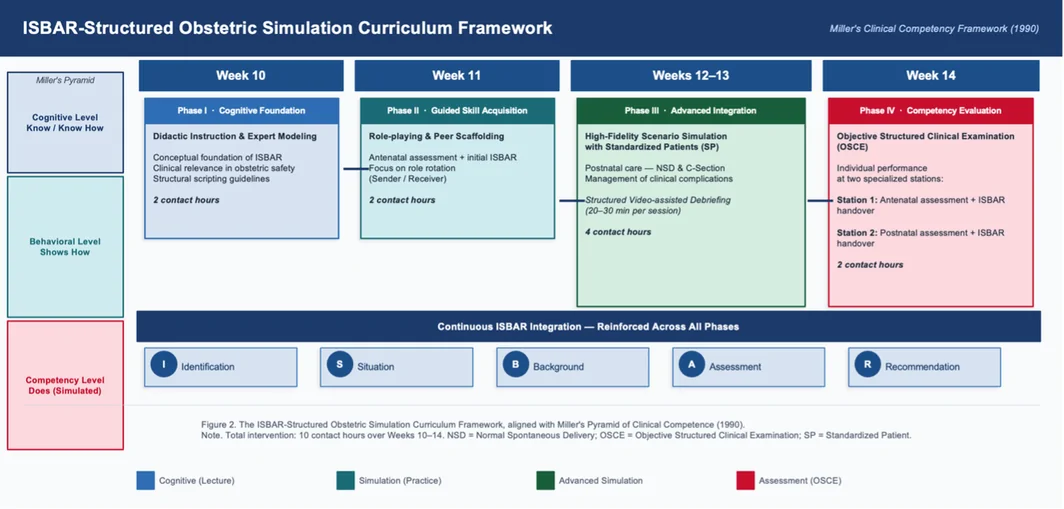
Intervention flow. The ‘2 contact hours’ shown for Phase IV (OSCE) denote scheduled student contact time; the total assessment operating time is reported in Section 2.4.

### 2.5 Research Instruments

#### 2.5.1 Quantitative Instruments

(1) Demographic questionnaire: age, gender and prior experience with ISBAR handover and OSCE.

(2) Communication Clarity Scale (CCS): This study adopted the CCS revised by Cho (2013) from Marshall et al.'s (2009) scale to assess the clarity of nursing students' handover content. The scale comprises 14 items scored on a five‐point Likert scale (1 = strongly disagree to 5 = strongly agree), with total scores ranging from 14 to 70; higher scores indicate greater communication clarity. The overall Cronbach's α for internal consistency was 0.77.

(3) Confidence Scale: The Grundy (1993) Confidence Scale was used to measure nursing students' degree of confidence in clinical handover at pre‐ and post‐test. The scale comprises five items scored on a five‐point Likert scale (1 = strongly disagree to 5 = strongly agree), with scores ranging from 5 to 25; higher scores indicate greater confidence. The overall Cronbach's α for internal consistency was 0.84.

(4) OSCE scores: Scoring rubrics for both stations (maximum 30 points, converted to a percentage scale) and OSCE case scenarios were co‐developed and reviewed by five experts (the researcher, head nurse of the delivery room, head nurse of the postpartum unit, an obstetric nurse with 5 years' experience and a postpartum nurse with 5 years' experience) based on hospital ISBAR handover standards and clinical training cases for new staff. Each station carried a maximum of 30 points, converted to a percentage scale. All raters participated in a joint scoring calibration exercise prior to the formal OSCE to ensure inter‐rater consistency. Inter‐rater reliability was quantified by independent double‐scoring: a randomly selected subset of 24 students had both OSCE stations independently scored by both assessors, yielding 48 of 114 total station performances (42.1%) scored by both raters; the two‐way random‐effects, absolute‐agreement, average‐measures intraclass correlation coefficient, ICC (2, k), was 0.82, 95% CI [0.71, 0.90] for Station 1 and 0.83, 95% CI [0.72, 0.91] for Station 2 (Koo and Li 2016). For double‐scored performances, the final OSCE score used in analysis was the mean of the two raters' scores; all remaining performances were scored by a single trained assessor. The OSCE was conducted at the end of the program (Week 14) as the final stage of the four‐phase design. Double‐scoring was performed by two designated assessors (the same two throughout): for the 24 double‐scored students, both were present in the same station room, observed each performance at the same time and recorded their scores independently without conferring; during these slots, the room vacated by the second assessor was paused, while the remaining two assessors continued single‐scoring in their own rooms. This arrangement lengthened the total assessment operating time (Section 2.4). Supplementary S2 provides a summary of the scoring rubric, including its assessment domains and scoring criteria for both stations.

(5) Learning satisfaction questionnaire: An online scenario simulation satisfaction questionnaire comprising five rated items and two open‐ended questions. Rated items used a five‐point Likert scale (1 = strongly disagree to 5 = strongly agree); all responses were anonymous. The five items assessed satisfaction with course content, teaching activities, learning outcomes, simulation authenticity and overall learning experience. The two open‐ended items collected students' qualitative feedback.

#### 2.5.2 Qualitative Instruments

(1) Learning reflection feedback forms and researcher's teaching reflection records.

(2) Focus group interviews: Semi‐structured interviews were conducted with volunteer students after the course (Week 18), after all course assessments were completed and final grades released, and each interview lasted approximately 40–50 min.

### 2.6 Data Collection

Pre‐test (Week 1): Following IRB approval, the study was explained to students and consent forms were collected before the formal course commenced, the CCS and confidence scale were administered. The 5‐week ISBAR‐structured simulation teaching program was implemented in Weeks 10–14, and the OSCE and post‐test scales were administered in Week 14. Recruitment e‐mails were sent within 1 week of the program ending, but the interviews were conducted in Week 18, after all course assessments were completed and final grades released, so that no student was interviewed while their grade remained pending. Participants were divided into four focus groups of 6–7 persons each, totaling 26 participants. Each group participated in one interview session (no repeat interviews were conducted) lasting approximately 40–50 min. The demographic characteristics of the 26 interview participants were not tabulated separately; therefore, their comparability with the full cohort (Table 1) cannot be established, and volunteers may have differed from non‐volunteers (see Section 5.1). The focus‐group interviews were conducted in a discussion room within the Department of Nursing. Supplementary S3 provides an interview questioning route. Interviews were facilitated using a semi‐structured interview guide by a female nurse with a doctoral degree (J.C.S.), who served as the principal course instructor during the research period and holds a faculty position in the nursing department with training in focus group facilitation and experience in qualitative research teaching. As the principal course instructor, J.C.S. had an established teacher–student relationship with participants prior to the study; participants had also been informed, at the time of consent, that the purpose of the research was to evaluate and improve ISBAR‐structured handover teaching within the course. To minimize the influence of the instructor's dual role as facilitator, an assistant facilitator with no teaching role attended each session; no other observers were present. Participants were informed of the study purpose, procedures and audio‐recording usage, ensuring that their rights were not affected. Open‐ended questions were used to facilitate group discussion, gather learning experiences and encourage diverse perspectives; the assistant facilitator simultaneously recorded participants' non‐verbal expressions and prompted for dissenting views. Because the interviews were facilitated by the course instructor, they were scheduled only after grades were finalized, and participants were told that nothing said could affect their grades. The interview guide explicitly invited criticism, and transcripts were anonymized before independent coding by three authors, two of whom had no teaching or grading relationship with participants (see Section 2.7). Residual social‐desirability bias cannot be excluded. Interviews continued until data saturation was achieved, the research team conducted an immediate post‐session review to support subsequent analysis.

**TABLE 1:** Participant demographic characteristics (*N* = 57).

Variable	Mean (range)	*n*	%
Age (years)	20.30 (20–21)		
Sex			
Male		6	10.5%
Female		51	89.5%
Prior ISBAR handover lecture			
Yes		21	36.8%
No		36	63.2%
Prior ISBAR handover simulation teaching			
Yes		3	5.3%
No		54	94.7%
Prior OSCE experience			
Yes		57	100%
No		0	0%

*Note:* Participants were third‐year undergraduate nursing students enrolled in the Obstetric Simulation and Practice elective at a technology university in northern Taiwan. All participants had previously completed OSCE as part of their second‐year medical‐surgical nursing course.

Abbreviations: ISBAR = Introduction–Situation–Background–Assessment–Recommendation, OSCE = Objective Structured Clinical Examination.

### 2.7 Data Analysis

Quantitative data: SPSS 28.0 software was used for statistical analysis. Analytical methods included means, standard deviations, percentages and paired‐sample *t*‐tests. Normality of the pre–post difference scores was examined using the Shapiro–Wilk test (communication clarity *W* = 0.964, *p* = 0.083; confidence *W* = 0.982, *p* = 0.537), supporting the use of paired‐samples *t*‐tests. Mean differences with 95% confidence intervals and within‐subject effect sizes (Cohen's *d*_z_ = *t*/√*n*) are reported for all comparisons (Cohen 1988). Because the 14 communication‐clarity and five confidence items were tested separately, Bonferroni‐adjusted alphas of 0.0036 and 0.010 were applied; item‐level results are exploratory. Qualitative data: Audio recordings from the focus group interviews were transcribed into 87 pages of text, and 57 reflective journals were collected. Data were analysed using content analysis (Mayring 2021). Coding was conducted independently by the first author (J.C.S.), a co‐author (M.Y.C.) and the corresponding author (Y.C.T.). J.C.S. was the course instructor and interview facilitator, whereas M.Y.C. and Y.C.T. had no teaching or grading relationship with the participants. To limit the influence of the instructor's dual role, coding began only after final grades had been released, all three coders worked from anonymized transcripts, and the independent codes of the two non‐teaching coders served as a check on J.C.S.'s interpretations; codes were retained only when agreed through consensus. Following initial concept formation, all authors collectively discussed the appropriateness of the analytical findings based on their observational records and verbatim transcripts of reflective discussions. The research team held regular meetings to resolve coding discrepancies, clarify coding‐related issues and refine codes as necessary through consensus. These strategies were employed to enhance the rigour of the qualitative component of the research design. The de‐identification procedure followed three steps. First, before coding, each transcript was de‐identified by replacing participants' names with codes (e.g., Focus Group 1, Participant 3); the file linking these codes to participants' contact details was stored separately from the transcripts and was not used during coding. Second, this linkage file was used solely to re‐contact participants for preliminary category checking. Third, the linkage file was destroyed after preliminary category checking was completed. To examine the credibility of the findings, member checking was conducted at a single point: during category development, six participants who remained contactable verified the accuracy and resonance of the preliminary categories. Member checking of the final confirmed findings was not undertaken, because the linkage file had been destroyed after preliminary category checking, so participants could no longer be identified or re‐contacted. Verbatim transcripts themselves were not returned to participants for review or correction; the member‐checking process described above pertained to preliminary coded categories rather than raw transcripts. Coded transcripts were subsequently imported into NVIVO 12 for analysis. Data analysis continued until data saturation was reached—that is, until no new relevant codes emerged from the data. Supplementary S4 presents an example of the coding of transcribed data from meaning units. The resulting themes and subthemes were integrated using a side‐by‐side joint display, categorized as agreement, partial agreement, dissonance or silence (McCrudden et al. 2021; O'Cathain et al. 2010). The qualitative component is reported in accordance with the COREQ checklist (Tong et al. 2007); the completed checklist is provided as Supplementary S5.

### 2.8 Ethical Considerations

This study was approved by the Institutional Review Board of Landseed International Hospital (No.: IRB‐24‐009‐B1) and conducted with the consent of the participating university. Prior to participation, all participants were fully informed and provided written informed consent, indicating their clear understanding of the study purpose, procedures, data collection methods and principles of confidentiality and anonymity. Participation was entirely voluntary and unrelated to course requirements or academic performance. Participants were free to withdraw at any time without any adverse consequences. All research data were coded to ensure anonymity and data security.

## 3 Results

### 3.1 Demographic Characteristics

A total of 57 students participated in this study: 6 male (10.5%) and 51 female (89.5%), with a mean age of 20.30 years. Regarding prior ISBAR experience, 21 students (36.8%) had previously received ISBAR handover instruction and 36 (63.2%) had not; 3 (5.3%) had received ISBAR handover scenario teaching and 54 (94.7%) had not. All 57 students (100%) had prior OSCE experience, as second‐year students in the four‐year nursing program participate in an OSCE at the end of their medical‐surgical nursing course (Table 1).

### 3.2 Communication Clarity

Following the ISBAR‐structured handover simulation intervention, students' overall post‐test scores on the Communication Clarity Scale (mean = 63.47, SD = 6.32) significantly exceeded pre‐test scores (mean = 51.24, SD = 11.43) (*t* = 7.87, *p* < 0.001; mean difference 12.23, 95% CI [9.12, 15.34], Cohen's *d*_z_ = 1.04). Post‐test mean scores on all 14 items across the five domains—Introduction, Situation, Background, Assessment and Recommendation—exceeded pre‐test means and demonstrated statistically significant differences (all *p* < 0.001 except ‘State the location’, *p* = 0.002; all remained significant under the Bonferroni‐adjusted alpha of 0.0036), indicating higher communication clarity after the intervention, though this improvement cannot be attributed solely to the program (Table 2).

**TABLE 2:** Pre‐ and post‐intervention differences in communication clarity among nursing students (*N* = 57).

Item	Pre‐test M (SD)	Post‐test M (SD)	*t*	*p*	Mean difference [95% CI]	Cohen's *d*_z_
Total—Communication Clarity Scale	51.24 (11.43)	63.47 (6.32)	7.87	**< 0.001**	12.23 [9.12, 15.34]	1.04
Introduction (I)						
Introduce yourself (name)	4.33 (0.95)	4.86 (0.35)	4.04	**< 0.001**	0.53 [0.27, 0.79]	0.53
Introduce your role (nursing student)	4.40 (0.90)	4.86 (0.35)	3.71	**< 0.001**	0.46 [0.21, 0.71]	0.49
State the location (ward/bed)	4.33 (0.93)	4.77 (0.46)	3.25	**0.002**	0.44 [0.17, 0.71]	0.43
Confirm the other party's identity	4.44 (0.88)	4.88 (0.33)	3.73	**< 0.001**	0.44 [0.20, 0.68]	0.49
Situation (S)						
Briefly state the purpose of handover	3.26 (1.11)	4.46 (0.68)	7.50	**< 0.001**	1.20 [0.88, 1.52]	0.99
State the urgency or appropriate concern	3.11 (1.12)	4.30 (0.75)	8.01	**< 0.001**	1.19 [0.89, 1.49]	1.06
Clearly emphasize the patient's problem and urgency	3.21 (1.06)	4.30 (0.70)	7.32	**< 0.001**	1.09 [0.79, 1.39]	0.97
Convey problems in key points and logical order	3.11 (1.12)	4.32 (0.73)	7.77	**< 0.001**	1.21 [0.90, 1.52]	1.03
Background (B)						
State the patient's medical information	4.04 (1.06)	4.60 (0.65)	3.64	**< 0.001**	0.56 [0.25, 0.87]	0.48
Assessment (A)						
State critical vital signs	3.93 (0.97)	4.63 (0.55)	4.67	**< 0.001**	0.70 [0.40, 1.00]	0.62
Briefly and accurately summarize the main problems	3.44 (0.98)	4.46 (0.60)	7.36	**< 0.001**	1.02 [0.74, 1.30]	0.98
Clearly describe the required treatment for this condition	3.28 (1.06)	4.40 (0.67)	7.80	**< 0.001**	1.12 [0.83, 1.41]	1.03
Recommendation (R)						
Directly seek help or make a recommendation	3.25 (1.10)	4.35 (0.76)	6.46	**< 0.001**	1.10 [0.76, 1.44]	0.86
Raise specific questions or clarify instructions	3.12 (0.98)	4.30 (0.70)	8.04	**< 0.001**	1.18 [0.89, 1.47]	1.06

*Note:* CCS = Communication Clarity Scale (Cho 2013, adapted from Marshall et al. (2009)). Items scored on a 5‐point Likert scale (1 = strongly disagree to 5 = strongly agree); total range 14–70. Cohen's *d*_z_ = *t*/√*n*; 0.20 = small, 0.50 = medium, 0.80 = large (Cohen 1988). Item‐level analyses are exploratory; all remained significant under the Bonferroni‐adjusted alpha of 0.0036. Two‐tailed paired‐samples *t*‐test. All values are presented as Mean ± SD. *p*‐values are derived from paired‐samples *t*‐tests comparing pre‐intervention and post‐intervention scores. *** *p* < 0.001.

Abbreviations: A = Assessment, B = Background, CI = confidence interval for the mean difference, df = 56. I = Introduction, *R* = Recommendation, S = Situation.

### 3.3 Handover Confidence

Following the ISBAR‐structured handover simulation intervention, students' overall post‐test scores on the Confidence Scale (mean = 19.15, SD = 3.53) significantly exceeded pre‐test scores (mean = 13.64, SD = 3.69) (*t* = 10.63, *p* < 0.001; mean difference 5.51, 95% CI [4.47, 6.55], Cohen's *d*_z_ = 1.41). Post‐test mean scores on all five items of the Confidence Scale exceeded pre‐test means and demonstrated statistically significant differences (*p* < 0.001; all remained significant under the Bonferroni‐adjusted alpha of 0.010), indicating higher handover confidence after the intervention (Table 3).

**TABLE 3:** Pre‐ and post‐intervention differences in handover confidence among nursing students (*N* = 57).

Item	Pre‐test M (SD)	Post‐test M (SD)	*t*	*p*	Mean difference [95% CI]	Cohen's *d*_z_
Total—Confidence Scale	13.64 (3.69)	19.15 (3.53)	10.63	**< 0.001**	5.51 [4.47, 6.55]	1.41
My performance is correct	2.75 (0.83)	4.02 (0.61)	11.25	**< 0.001**	1.27 [1.04, 1.50]	1.49
I feel able to complete the task independently	2.51 (0.84)	3.74 (0.76)	6.86	**< 0.001**	1.23 [0.87, 1.59]	0.91
My performance makes the observer confident I can handle this task	2.79 (0.90)	3.74 (0.93)	7.55	**< 0.001**	0.95 [0.70, 1.20]	1.00
I feel confident in myself while performing the task	2.75 (0.85)	3.75 (0.85)	8.24	**< 0.001**	1.00 [0.76, 1.24]	1.09
I am satisfied with my own performance	2.84 (0.70)	3.91 (0.93)	9.06	**< 0.001**	1.07 [0.83, 1.31]	1.20

*Note:* Confidence Scale (Grundy 1993). Items scored on a 5‐point Likert scale (1 = strongly disagree to 5 = strongly agree); total range 5–25. Cohen's *d*_z_ = *t*/√*n*; Item‐level analyses are exploratory; all remained significant under the Bonferroni‐adjusted alpha of 0.010. Two‐tailed paired‐samples *t*‐test, df = 56. All values are presented as Mean ± SD. *p*‐values are derived from paired‐samples *t*‐tests comparing pre‐intervention and post‐intervention scores. *** *p* < 0.001.

Abbreviation: CI = confidence interval for the mean difference.

### 3.4 OSCE Results

After implementing ISBAR‐structured handover didactic instruction and expert modelling, role simulation and scenario simulation, students completed two OSCEs: OSCE 1‐‘Antenatal Assessment and ISBAR‐Structured Handover’; and OSCE 2‐‘Normal Spontaneous Delivery Postpartum Assessment and ISBAR‐Structured Handover’. All students were required to participate in the ISBAR‐structured handover simulation teaching and both OSCEs. Antenatal assessment and ISBAR handover: mean score = 87.75, SD = 5.23; normal spontaneous delivery postpartum assessment and ISBAR handover: mean score = 87.26, SD = 5.24 (Figure 3).

**FIGURE 3:**
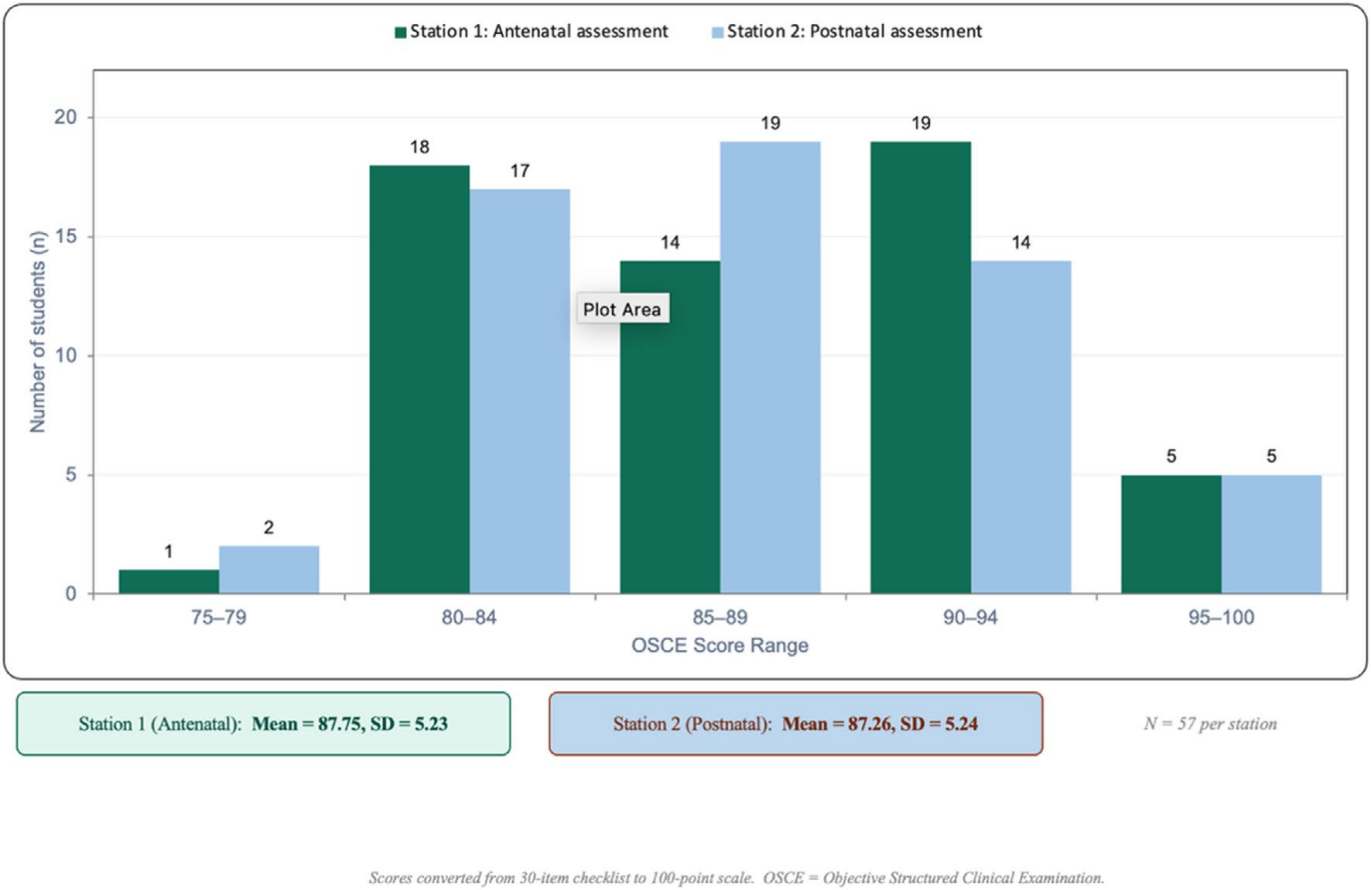
OSCE score distribution.

### 3.5 Learning Satisfaction

Scored on a five‐point Likert scale; higher scores indicate greater satisfaction with the course (Table 4).

**TABLE 4:** Learning satisfaction (*N* = 57).

Item	Mean score	SD
The course content aligns with the teaching objectives	4.92	0.28
The teaching methods facilitated my learning	4.91	0.34
The teaching activities facilitated my learning	4.92	0.32
I engaged seriously in this course	4.90	0.36
Overall, this course was highly beneficial to me	4.92	0.29

*Note:* Items rated on a 5‐point Likert scale (1 = strongly disagree to 5 = strongly agree). Higher scores indicate greater satisfaction. Item means were close to the scale maximum with minimal dispersion, indicating a pronounced ceiling effect; these data are reported descriptively only. All responses were collected anonymously via an online platform.

Word cloud analysis of students' open‐ended responses before (Figure 4) and after (Figure 5) the clinical handover intervention revealed that, prior to the intervention, students described feelings of nervousness and uncertainty when questioned; following the intervention, responses showed markedly greater structure and confidence. These changes in language provide preliminary qualitative evidence that resonates with the two quantitative findings above—improvements in CCS scores and confidence—and contextualize the four thematic findings from the interview analysis presented below.

**FIGURE 4:**
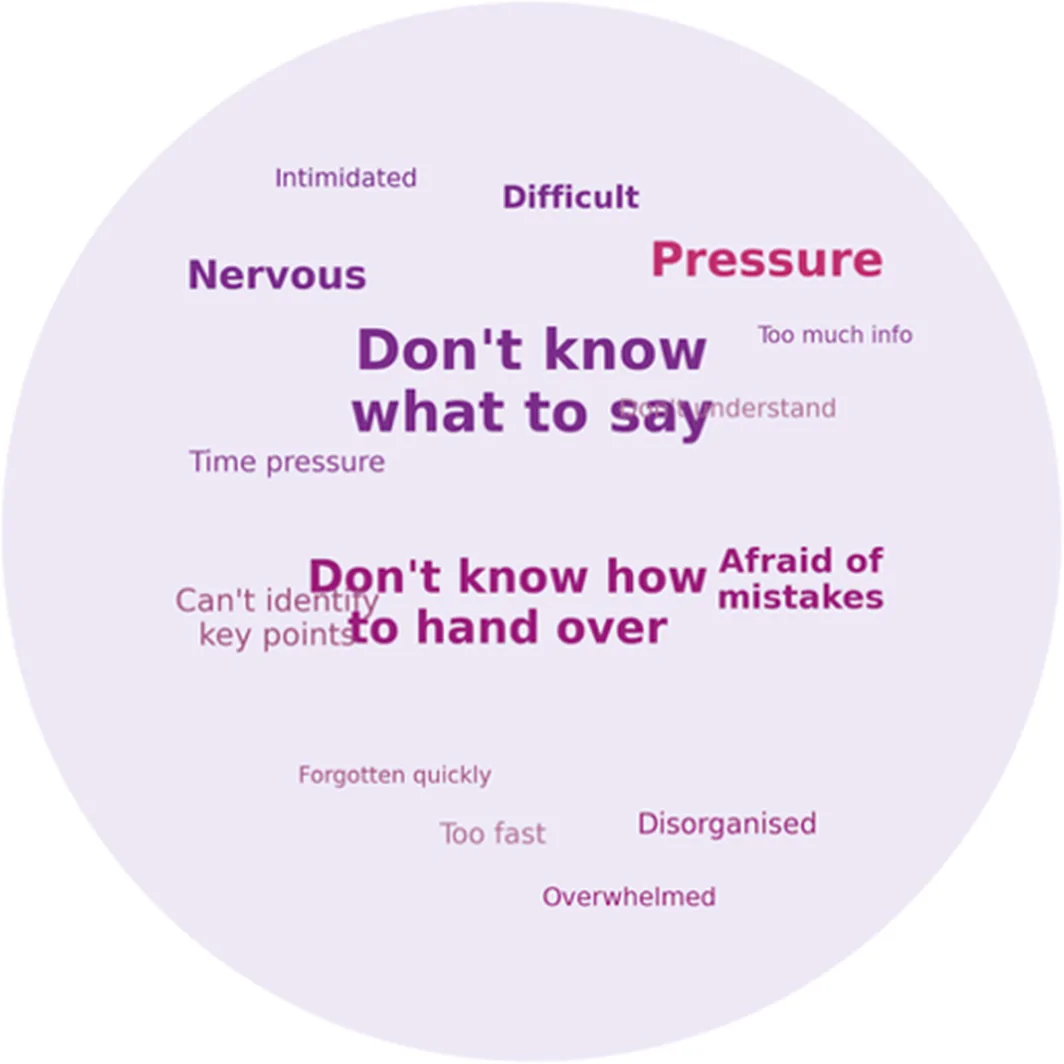
Word cloud of students' perception of clinical handover before intervention.

**FIGURE 5:**
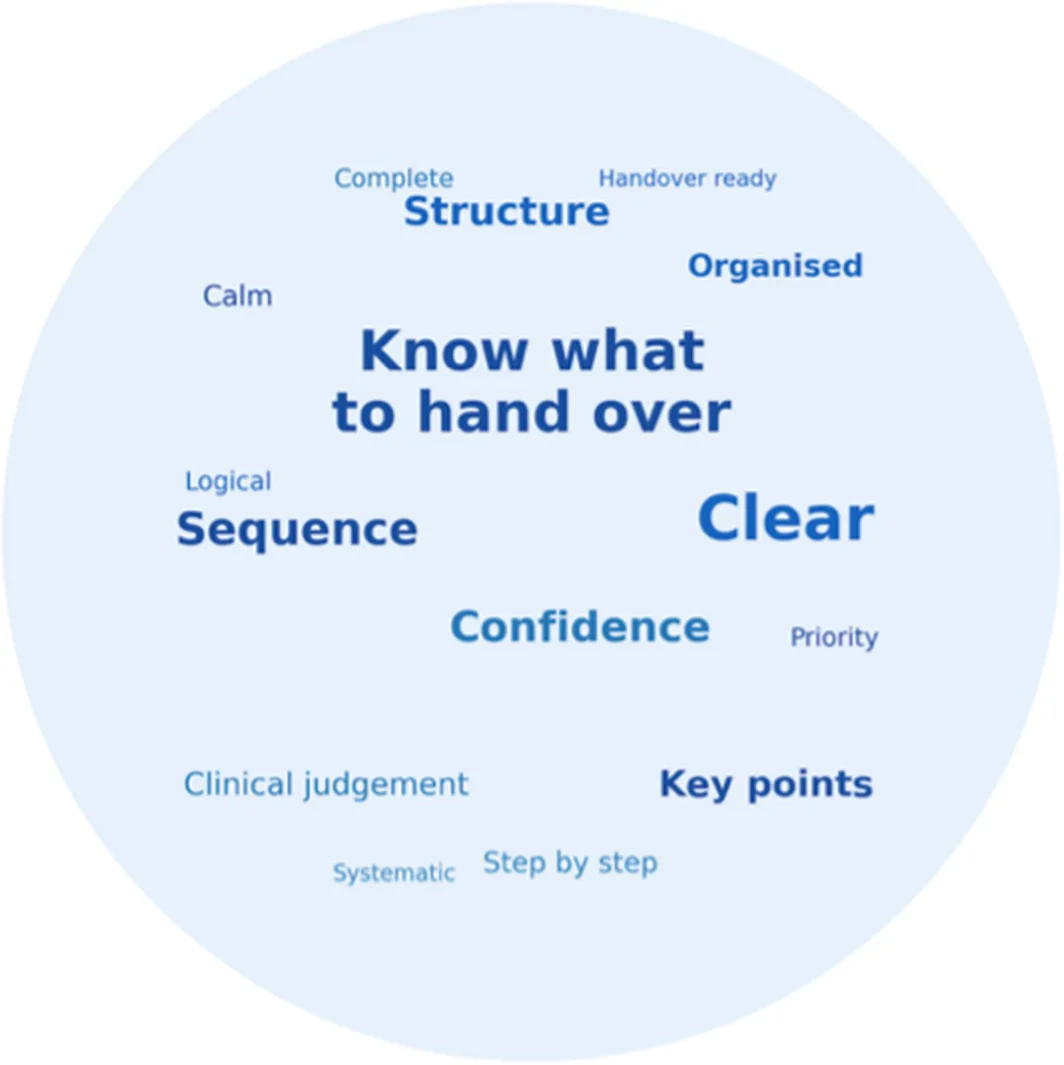
Word cloud of students' perception of clinical handover after intervention.

### 3.6 Learning Reflection, Feedback Forms and Focus Group Interview Analysis

#### 3.6.1 Mastering the Handover Framework to Enhance Organized Expression

Students generally reported that ISBAR provided a clear handover procedure, enabling them to organize and convey information more systematically, reducing the confusion and omissions they had previously experienced. Most students indicated that, when observing handovers during clinical placements, they had often been unable to identify key points; following this course, they could clearly distinguish the sequence and priorities of handover content.

Before, I would just say whatever came to mind, and the order was chaotic. Using ISBAR, I know to introduce myself and the patient first, then describe the patient's condition and background, then convey the important assessment and recommendations. The handover has structure, and the person receiving it can understand much more clearly (Focus group 1, Participant 3).

#### 3.6.2 Scenario Simulation Strengthening Clinical Reasoning and Adaptability

The antenatal and postpartum simulation scenarios arranged in the course facilitated the application of knowledge within simulated contexts. During practice, students were required to integrate assessment information in real time and conduct handovers according to the patient's condition, enabling them to practice integrating clinical information and responding immediately within the scenario.

In the simulation scenario, there were situations to assess and report; I had to think on the spot about how to supplement the information, and this helped me respond more flexibly (Reflective Journal, Participant 2).

#### 3.6.3 Peer Feedback and Reflection Promoting Self‐Awareness

Receiving peer and instructor feedback during the course helped students identify blind spots in their handover expression and subsequently refine their performance. In addition, completing written learning reflection forms facilitated self‐review of the learning process and areas for growth.

Watching my classmates’ handover, I would think about whether I have similar issues—like speaking too fast or not being clear enough (Reflective Journal, Participant 9).

#### 3.6.4 Progressive Practice Building Handover Confidence

The progressively structured learning design—from didactic instruction and role‐play through scenario simulation to OSCE—meant that repeated practice helped reduce students' unfamiliarity with and anxiety about handover, progressively building their confidence.

After each simulation, I improved; my handovers became clearer and more stable each time (Focus group 3, Participant 5).

Table 5 presents the integrated quantitative and qualitative results, mapping qualitative themes to corresponding quantitative findings and providing meta‐inferences from the mixed‐methods design. Across all four themes, quantitative and qualitative data consistently describe improvements observed after the intervention. As the design was uncontrolled and no follow‐up was undertaken, causal attribution and durability cannot be established. A minority of students, however, described persistent difficulty adapting to unscripted or unexpected events during scenario practice; this divergent pattern is examined further in the Discussion (Section 4).

**TABLE 5:** Joint display of quantitative and qualitative findings (*N* = 57; focus group *n* = 26).

Quantitative	Qualitative	Mixed‐methods meta‐inference
Theme 1: Mastering the handover framework to enhance organized expression CCS total score improved significantly Pre‐test: M = 51.24 (SD = 11.43) Post‐test: M = 63.47 (SD = 6.32) *t* = 7.87, *p* < 0.001	Mastering handover structure ‘The ISBAR process means I won't miss important information—like medical history, current status and follow‐up recommendations. The whole handover has structure’.—P7 ‘I used to jump around when I spoke. Now I organize information in the I–S–B–A–R sequence, and the logic is much clearer’.—P5	Complementarity Students reported that ISBAR provided a clear cognitive scaffold, reducing disorganization and omissions—directly convergent with CCS score improvements across all 14 items.
Theme 2: Scenario simulation strengthening clinical reasoning and adaptability OSCE Station 1 (Antenatal + ISBAR) M = 87.75 (SD = 5.23) OSCE Station 2 (Postnatal + ISBAR) M = 87.26 (SD = 5.24)	Simulation enhancing clinical thinking “The simulated cases were very similar to situations in real wards. I had to judge which information was most important to hand over.”—P8 “Because it simulated a clinical context, I would think: if I were the incoming nurse, what would I need to know? That trained my clinical judgement.”—P6	Convergence Post‐program OSCE performance was high and consistent across both scenarios, and students described real‐time judgement during simulation. As OSCE was measured only after the intervention, these sources describe the same end point rather than corroborating change.
Theme 3: Peer feedback and reflection promoting self‐awareness CCS items: communication clarity Pre‐test: M = 51.24 → Post‐test: M = 63.47 All 14 CCS items significant (13 items *p* < 0.001; ‘State the location’ *p* = 0.002) (including clarity, logical sequencing)	Peer feedback promoting self‐awareness “During role‐rotation practice, my classmates would tell me where I wasn't clear enough or had missed important information—that let me know my own problems.” — P1 “Watching my classmates' handover made me wonder whether I had similar problems, like speaking too fast or not clearly enough.” — P9 “My classmates' suggestions reminded me that some information I understood myself wasn't necessarily clear to the listener. I need to explain it more explicitly.” — P4	Expansion Quantitative data show improved communication clarity but cannot explain the mechanism, expanding on the qualitative findings.
Theme 4: Progressive practice building handover confidence Confidence scale improved significantly Pre‐test: M = 13.64 (SD = 3.69) Post‐test: M = 19.15 (SD = 3.53) *t* = 10.63, *p* < 0.001	Progressive practice building confidence “I was very nervous at first, afraid of saying something wrong or leaving things out. But after a few rounds of practice, I could hand over more calmly.”—P10 “Repeated practice made me more familiar with ISBAR. When I encounter real handover in clinical placement, I won't be so afraid.” — P7	Convergence The quantitative gain (confidence, *t* = 10.63) converges with qualitative accounts of graduated anxiety reduction across the four‐phase design (lecture → role‐play → simulation → OSCE).

*Note:* Qualitative themes derived from content analysis of focus group transcripts (87 pages), student reflective journals (*n* = 57) and researcher teaching reflections (Mayring 2021).

Abbreviations: CCS = Communication Clarity Scale, Divergence = findings are incongruent and require further investigation, Expansion = qualitative data extend or explain the quantitative findings, ISBAR = Introduction–Situation–Background–Assessment–Recommendation, M = mean, Meta‐inference categories: Convergence = quantitative and qualitative findings mutually confirm the same conclusion, OSCE = Objective Structured Clinical Examination, SD = standard deviation.

## 4 Discussion

The improvement in communication clarity observed after the intervention represents the most robust quantitative finding of this study. All 14 individual scale items showed statistically significant pre‐post differences, indicating that—under the complete ISBAR framework—improvement was broad in scope rather than confined to specific domains. The most notable finding within the five ISBAR components was the greatest improvement in the situation domain, particularly in items such as ‘concisely stating the purpose of the handover’ and ‘conveying urgency or appropriate concern’. This suggests that students developed not only structural memory but also greater capacity to discern which information is urgent in real‐world practice scenarios and to carry that forward into their communication. This distinction is noteworthy and supports the recommendation that scenario‐based simulation fosters clinical reasoning—an objective that didactic instruction alone is less capable of achieving. These results are consistent with and extend prior research. Noh and Park (2022) demonstrated significant improvement in nursing students' handover quality in a quasi‐experimental SBAR study; Kim et al. (2016) found that SBAR course participants demonstrated improved communication clarity and clinical competence. More recently, Vanderzwan et al. (2023) found that adherence to structured scenario simulation yielded significant post‐test improvement in handover accuracy (*p* < 0.001), closely aligning with the present findings. Crucially, those studies were conducted in general nursing contexts, whereas the present study extends the evidence base to obstetric nursing students. Obstetrics demands particular acuity in detecting changes in patient condition and time‐sensitive decision‐making, given that communication failures may result in serious consequences for both the mother and infant. These findings therefore suggest that ISBAR‐structured handover scenario simulation teaching should be considered for inclusion as a required component of obstetric nursing curricula.

The second core quantitative finding was a substantial improvement in handover confidence, with significant increases across all five subscale items. Examining this more closely, the magnitude of improvement in self‐efficacy is particularly notable, exceeding that observed for communication clarity. This suggests that students' beliefs in their own capability—a sense of psychological readiness—may be a prerequisite for translating acquired ISBAR knowledge into clinical practice. This is consistent with Kolb's (1984) experiential learning theory, whereby repeated simulation practice generates concrete experience, enabling students to progressively build competence as anxiety diminishes, and motivating more thorough engagement with subsequent reflective and abstract learning phases. One participant noted: ‘At first I was very nervous about handover, afraid of saying the wrong thing or missing something, but after practicing a few times, I could hand over in a much more relaxed way’ (P10). The integration of quantitative confidence gains and qualitative accounts of gradually diminishing anxiety reveals the mechanism underlying the improvement in confidence. These findings echo those of Kostiuk (2015) and Chung et al. (2022), who note that students lacking confidence may avoid handover or omit critical information under pressure, pre‐clinical simulation practice can effectively mitigate this risk. By helping students build confidence through simulation practice before entering clinical settings, the transition from classroom to ward may be achieved with a reduced risk of such behaviours.

The two OSCE stations (antenatal and postpartum ISBAR handover) yielded high mean scores with low variability, indicating consistent performance under standardized conditions at the end of the program; as OSCE was measured only after the intervention and without a comparison group, these scores document attained performance rather than improvement. These scores cannot be combined with the pre–post scale data to strengthen a causal inference. Burgess et al. (2020) argue that OSCE enables educators to obtain objective, criterion‐referenced data in controlled environments. The similarity of scores across the two stations indicates that performance was stable across two different obstetric scenarios. As both stations were drawn from content taught during the program, these scores should not be read as evidence of transferable competence.

The strengths of the mixed‐methods design lie not only in explaining what improved but also in illuminating why and how improvement occurred. Across all four themes, a consistent pattern emerged in which students transitioned from passive compliance to active and reflective clinical communication. The ISBAR framework initially provided a cognitive scaffold—organizing information through a reliable sequence to reduce the cognitive load of handover; through successive simulation practice, this scaffold became progressively internalized. Scenario simulation with standardized patients, consistent with Miller's (1990) framework, guided students in practicing authentic clinical judgement rather than scripted recall, and in demonstrating competence. The rotation of roles, video‐assisted debriefing and reflective journals created iterative cycles of practice, observation and self‐correction, deepening both structural proficiency and communicative flexibility—consistent with the findings of Chang et al. (2025) and Johnston et al. (2017). However, the verisimilitude constraints of simulation may have led some students to over‐rely on preset scripts and overlook clinical urgency; insufficient design of information updates and unexpected events may have caused some students to remain at the level of ‘completing the handover procedure’ rather than developing clinical decision prioritization and adaptability. The provision of immediate feedback and re‐practice opportunities helped students refine their performance, though linguistic fluency and in‐the‐moment clinical judgement still require further accumulation of experience.

## 5 Conclusion

This study is the first to employ a mixed‐methods design to examine changes in nursing students' clinical handover performance associated with ISBAR‐structured simulation teaching in obstetric nursing education. Quantitative findings showed significantly higher post‐intervention scores for handover clarity and confidence. Qualitative findings further revealed that students transitioned from passive to active and reflective communicators. The integration of self‐report scales, OSCE performance, and qualitative data provides a more comprehensive portrayal of the learning process and its outcomes than any single data source could afford. The findings support the incorporation of systematic, structured scenario‐based practice into nursing curricula, particularly in high‐acuity units where high‐quality handover is essential for patient safety.

### 5.1 Limitations

This study employed a single‐group pre‐post design and lacked a control group, making it impossible to exclude maturation and Hawthorne effects; moreover, the study was conducted at a single institution, limiting generalizability. The confidence scale instrument is somewhat dated and was not specifically designed for nursing students. In addition, the course instructor (J.C.S.) facilitated the focus groups and participated in coding; although interviews and coding took place after grades were finalized, transcripts were anonymized and two coders without a teaching relationship were involved, instructor influence on data generation and interpretation cannot be fully excluded. Interview participants were self‐selected volunteers (26 of 57), and because their demographic characteristics were not tabulated separately, their representativeness of the full cohort cannot be established. Patient‐safety outcomes were not measured; implications for patient safety are therefore inferential. It is recommended that further research adopt randomized controlled or quasi‐experimental designs and develop handover self‐efficacy measurement instruments that are culturally appropriate and specific to obstetric contexts.

### 5.2 Implications for Nursing

Nursing educators should consider incorporating ISBAR‐structured simulation practice into specialist nursing curricula, particularly in high‐risk areas such as obstetrics. As this study employed a single‐site, pre‐experimental design, the four‐stage sequence outlined below is offered as a potentially useful educational strategy rather than a validated model, and its effectiveness warrants further evaluation across different settings and populations before broader adoption. The development of handover competence should not rely on incidental practice opportunities during clinical placements; instead, planned, progressive development should be undertaken before clinical placement through a four‐stage design: didactic instruction, role‐play, scenario simulation and OSCE. This progressively structured model can serve as a flexible template for educators to adapt according to different specialist content. Furthermore, learning in a low‐risk professional environment helps students build a sense of achievement and internalize skills. Without structured practice, student nurses entering clinical settings are often anxious due to the presence of senior nurses or environmental pressures, leading them to avoid handover or omit critical information—behaviours that pose potential threats to patient safety (Le et al. 2023; Malone et al. 2016). Finally, nursing managers and clinical nurse educators should recognize that handover confidence and clarity are trainable outcomes. Incorporating brief ISBAR simulation reviews into new staff orientation programs can reduce skills attrition and support students during the transitional period upon entering clinical practice.

## Author Contributions

**Jui‐Chiung Sun:** conceptualization, methodology, investigation, formal analysis, writing – original draft, writing – review and editing, funding acquisition. **Min‐Yu Chang:** methodology, investigation, writing – review and editing, formal analysis. **Yi‐Chun Tsai:** conceptualization, methodology, formal analysis, validation, writing – review and editing.

## Funding

This research was funded by the Ministry of Education, MOE, grant number PED1135003.

## Disclosure

During the preparation of this manuscript, the authors used ChatGPT for English language editing. This tool did not collect, analyse or interpret any data, and all generated content was reviewed and finalized by the authors. All statistical values were verified against original statistical output by the authors. The authors reviewed, edited and verified all scientific content, interpretations, analyses and conclusions and assume full responsibility for the entirety of this publication. No artificial intelligence tool was listed as an author or holds any responsibility for this work.

## Ethics Statement

This study was approved by the Institutional Review Board of Landseed International Hospital (No.: IRB‐24‐009‐B1). Informed consent was obtained from all participants.

## Conflicts of Interest

The authors declare no conflicts of interest.

## Supporting information


**Supplementary S1:** OSCE timetable.

**Supplementary S2:** A summary of the scoring rubric, including its assessment domains and scoring criteria for both stations.

**Supplementary S3:** An interview questioning route.

**Supplementary S4:** An example of the coding of transcribed data from meaning units.

**Supplementary S5:** Consolidated criteria for reporting qualitative research (COREQ) 32‐item checklist.

## Data Availability

The data that support the findings of this study are available from the corresponding author upon reasonable request. The data are not publicly available because they contain information that could compromise the privacy of the participants.
