# Supplementary material for: Changes in Nursing Students' Clinical Handover Performance Following ISBAR‐Structured Handover Simulation Teaching: A Mixed‐Methods Study
Source: Nurs Open. 2026 Oct 26;13(10):e70955. doi: 10.1002/nop2.70955 (PMC13710796; doi:10.1002/nop2.70955)
Supplement: Supplementary file 2 [file NOP2-13-e70955-s001.docx]

Supplementary 2**.** A summary of the scoring rubric, including its assessment domains and scoring criteria for both stations.

## OSCE Station 1: Antenatal Assessment and ISBAR-Structured Handover

**Time limit: 10 minutes**

| Domain | Assessment content | Max points |
| --- | --- | --- |
| A. Introduction & Patient Identification | Self-introduction; verification of patient identity and explanation of the assessment purpose | 2 |
| B. Intrapartum Assessment | Dietary and voiding status; fetal heart beat interpretation; uterine contraction pattern (interval, duration, intensity); pain assessment (NRS); signs of impending second stage of labor; bloody show and rupture-of-membranes status | 8 |
| C. Patient Education | Education on rupture-of-membranes precautions; breathing techniques for contraction-pain relief | 4 |
| D. ISBAR-Structured Handover | **I**dentification (bed number, name, diagnosis); **S**ituation (obstetric history); **B**ackground (past history); **A**ssessment (vital signs; intrapartum findings — diet/voiding, fetal heartbeat, contractions, bloody show/rupture of membranes, cervical os/effacement/station; education provided); **R**ecommendation (education needs; current IV medication) | 13 |
| E. Overall Clinical Performance | Global rating of professionalism, communication clarity, and organization of care | 3 |
| **Total** |  | **30** |

## OSCE Station 2: Normal Spontaneous Delivery Postpartum Assessment and ISBAR Handover

**Time limit: 10 minutes**

| Domain | Assessment content | Max points |
| --- | --- | --- |
| A. Introduction & Patient Identification | Self-introduction; verification of patient identity and explanation of the postpartum assessment purpose | 2 |
| B. Postpartum Assessment (BUBBLE-HE) | **B**reast (inspection and palpation); **U**terus (fundal height and involution); **B**ladder (voiding status); **B**owel (dietary intake, food precautions, and bowel elimination); **L**ochia; **E**pisiotomy/perineum (REEDA assessment; perineal pain via PQRST); **H**oman’s sign; **E**motional status | 11 |
| C. ISBAR-Structured Handover | **I**dentification (bed number, name, diagnosis); **S**ituation (obstetric history); **B**ackground (past history); **A**ssessment (vital signs; postpartum findings — breast/uterus, bladder/bowel, lochia, perineal wound and pain, Homan’s sign, emotional status); **R**ecommendation (further perineal pain reassessment; current medication) | 14 |
| D. Overall Clinical Performance | Global rating of professionalism, communication clarity, and organization of care | 3 |
| **Total** |  | **30** |
