# Supplementary material for: Changes in Nursing Students' Clinical Handover Performance Following ISBAR‐Structured Handover Simulation Teaching: A Mixed‐Methods Study
Source: Nurs Open. 2026 Oct 26;13(10):e70955. doi: 10.1002/nop2.70955 (PMC13710796; doi:10.1002/nop2.70955)
Supplement: Supplementary file 3 [file NOP2-13-e70955-s002.docx]

Supplementary 3. An interview questioning route.

| Question | Probing question |
| --- | --- |
| Opening question | Please share your name and talk about your feelings or thoughts on "handover". |
|  |  |
| Main question | In the simulation, how does the ISBAR framework affect how you organize and present handover content?  What challenges did you encounter during the simulation? How did you deal with them?  After participating in the ISBAR structured handover simulation course, what specific suggestions do you have for this teaching method? |
|  |  |
| Ending question | Do you have any particularly insightful or worthwhile experiences to share during this course?  Is there anything else you feel should be discussed, but which we haven't mentioned? |
