# Supplementary material for: Changes in Nursing Students' Clinical Handover Performance Following ISBAR‐Structured Handover Simulation Teaching: A Mixed‐Methods Study
Source: Nurs Open. 2026 Oct 26;13(10):e70955. doi: 10.1002/nop2.70955 (PMC13710796; doi:10.1002/nop2.70955)
Supplement: Supplementary file 4 [file NOP2-13-e70955-s003.docx]

**Supplementary 1. OSCE timetable**

| **Item** | **Arrangement** |
| --- | --- |
| Scheduled student contact time (Figure 2) | 2 hours allocated to Phase IV in the course schedule; each student examined for 20 minutes (two 10-minute slots) |
| Class A (n = 32) – operating time | Week 14, first OSCE day, 12:30–17:30 (approximately 5 hours) |
| Class B (n = 25) – operating time | Week 14, second OSCE day, 12:30–16:30 (approximately 4 hours) |
| Rooms and assessor allocation | Four rooms in parallel (two Antenatal Assessment, two Postnatal Assessment); four trained assessors, one per room, scoring in real time with the same rubric; one trained standardized patient per room |
| Slot length | 10 minutes including changeover (each performance typically 7–8 minutes) |
| Double-scoring | 24 randomly selected students × 2 stations = 48 performances; the two designated assessors (the same two throughout) observed each performance together in one room and scored independently without conferring; the room vacated by the second assessor was paused, while the other two assessors continued single-scoring; 48 sequential slots = 480 minutes of joint assessor time across both sessions |
