# Supplementary material for: Changes in Nursing Students' Clinical Handover Performance Following ISBAR‐Structured Handover Simulation Teaching: A Mixed‐Methods Study
Source: Nurs Open. 2026 Oct 26;13(10):e70955. doi: 10.1002/nop2.70955 (PMC13710796; doi:10.1002/nop2.70955)
Supplement: Supplementary file 5 [file NOP2-13-e70955-s004.docx]

Supplementary 4. An example of the coding of transcribed data from meaning units.

Table S4-1 Example of the Coding of Transcribed Data from Meaning Units

| Meaning unit | Condensed meaning unit | Codes | Subtheme |
| --- | --- | --- | --- |
| " Using ISBAR during shift handover ensures I don't miss important information, such as medical history, current condition, and follow-up recommendations, giving the entire handover a more organized feel... " | ISBAR helps avoid missing important information and ensures that handover content is organized in sequence. | Improved information completeness  Structured handover process | Structured processes ensure information integrity |

Table S4-2 Themes and Subthemes resulting from the research

| Themes | Subthemes |
| --- | --- |
| Mastering the handover framework to enhance organized expression. | Structured processes ensure information integrity  Information reorganization improves the clarity of expression |
|  | Shifting to receiver-oriented professional communication |
|  |  |
| Scenario simulation strengthening clinical reasoning and adaptability | Priority judgment of contextual authenticity reinforcement |
|  | Real-time integration and dynamic response |
|  | Role transformation deepens professional identity |
|  |  |
| Peer feedback and reflection promoting self-awareness  Progressive practice building handover confidence | Peer feedback reveals blind spots  Writing reflections deepens self-awareness  Repeated drills reduce anxiety and build confidence  Constructive debriefing strengthening self-efficacy |
